# Integrated gut microbiota and metabolomics analysis of the effect of pomegranate peel powder on intestinal inflammation in broilers induced by high non-starch polysaccharides

**DOI:** 10.3389/fimmu.2026.1857398

**Published:** 2026-08-12

**Authors:** Fuwei Zhao, Lin Yi, Changming Zhou, Lili Jiang, Shiyu Wang, Xianlin Zhao, Shihao Ge, Jianbin Zhang, Zhaobin Fan

**Affiliations:** 1 College of Pharmacy, Heze University, Heze, China; 2 College of Animal Science and Veterinary Medicine, Tianjin Agricultural University, Tianjin, China

**Keywords:** inflammation, metabolites, microbiota, non-starch polysaccharides, pomegranate peel powder

## Abstract

**Background:**

Non-starch polysaccharides (NSPs) support gut health at low levels but may impair nutrient absorption and trigger inflammation when consumed excessively. Pomegranate peel powder (PPP), rich in punicalagin, ellagic acid, gallic acid, and quercetin, exerts intestinal protective effects by inhibiting pathogens, protecting probiotics, reducing inflammation, and strengthening the intestinal barrier. It shows potential for alleviating conditions such as infectious diarrhea, colitis, and metabolic intestinal disorders.

**Methods:**

This study investigates the mechanisms by which PPP mitigates NSP-induced intestinal inflammation in broilers. An inflammation model was established using a basal diet supplemented with 30% rice bran, validated through reduced growth performance, altered intestinal morphology, and increased inflammatory marker expression. Broilers receiving the high-NSP diet were supplemented with varying concentrations of PPP. An optimal dose of PPP (6 g/kg) was identified based on improvements in growth performance, intestinal inflammatory factor expression levels, and intestinal morphology. Further evaluation of this dose was carried out using 16S rRNA gene sequencing and LC-MS to evaluate its effect on the intestinal microbiota and fecal metabolites.

**Results:**

PPP supplementation significantly increased the relative abundance of Proteobacteria, Phascolarctobacterium faecium, Bacteroides uniformis, and Muribaculaceae, while reducing Firmicutes, Fusobacteriota, and Fusobacterium mortiferum. Metabolomic profiling identified 92 differential metabolites between PPP supplementation group and inflammation model group. PPP supplementation restored 25 metabolites change induced by NSPs, including geniposidic acid, succinic acid and 2,6-Dimethoxy-1,4-benzoquinone. KEGG analysis showed that oxidative phosphorylation, phenylalanine metabolism, and carbon metabolism are the most significantly enriched pathways.

**Conclusion:**

PPP could alleviate the inflammatory response and intestinal damage induced by NSPs, and promote the growth of broilers by strengthening the intestinal barrier and reducing the levels of inflammatory factors, as well as improving the intestinal flora and key functional metabolites.

## 1 Introduction

Broiler intestinal inflammation, one of the most harmful diseases, could lead to slower growth and lower feed conversion rate of chicken, causing high risk of infection and high cost of farming (1). Numerous previous studies have identified several factors that may induce gastrointestinal inflammation in broilers. These include high stocking densities, reused bedding material, the presence of intestinal pathogens such as *Eimeria* spp., substandard feed ingredients, diets rich in animal proteins, and elevated levels of NSPs (2, 3). NSPs are complex carbohydrates that are not broken down by digestive enzymes. They can significantly improve intestinal viscosity by combining with water to form gel network, resulting in decreased mixing efficiency of digestive enzymes and substrates, and decreased absorption rate of protein, fat and carbohydrate (4). Long-term intake will lead to intestinal villi atrophy, reduced expression of tight junction protein, and increased risk of intestinal leakage (5). NSPs are found in a wide variety of grains, such as barley, oats, wheat, rice bran and so on. Compared with other ingredients, such as wheat, rice provides higher metabolizable energy and less fiber. Rice bran is a by-product of rice milling. Owing to its high nutritional value and low-cost, rice bran has become a common cheap feed ingredient in poultry nutrition across rice-producing countries (e.g., Bangladesh, China, and India) (6). However, it is a typical NSP-rich ingredient (14.2% fiber, 1.37% phytate), and could induce oxidative damage to the intestinal mucosa during prolonged consumption (2, 3). Therefore, incorporating rice bran into diets might induce intestinal stress, and the severity of this effect is likely to depend on the level of rice bran inclusion. Recently, a feasible and easily replicated research dietary model of low-grade chronic inflammation in chickens was established with 30% rice bran (3).

Pomegranate peel, a by-product produced during pomegranate juice extraction, is readily accessible at low cost. Importantly, the skin of pomegranate is rich in high-molecular-weight phenols, which consists of tannins, procyanidins, and other complex polysaccharides (7, 8). Especially, tannins (i.e., punicalin, punicalagin, ellagic acid, and gallic acid) are the major components in the pomegranate peel extract. Punicalagin (28.03-104.14 mg/g), and ellagic acid (1.58-3.69 mg/g) are the dominant tannins in pomegranate peel extract, based on nine cultivars from seven major production areas in China (9). These compounds possess potential health benefits, including lipid-lowering effects, antioxidant activity, antitumor properties, anti-inflammatory action, and antibacterial effects (10). In addition, pomegranate peel serves as a significant reservoir of organic acids such as citric, malic, acetic, oxalic, tartaric, lactic, ascorbic, and fumaric acids, along with numerous other essential nutrients (11). It has been demonstrated that organic acids possess the capacity to reduce the pH level in the digestive tract, thereby engendering an environment that is more acidic. This acidification has been demonstrated to enhance the chicken’s ability to resist pathogens and to restrain the growth of specific intestinal bacteria, such as *E. coli*, which struggle to thrive in the low pH conditions (12). Research indicates that birds infected with *Eimeria* and given 6 g/kg pomegranate peel supplementation exhibited increased growth performance, reduced lesion severity, decreased oocyst excretion, and improved intestinal histological features (13).

The gut microbiota critically regulates host metabolism via microbial metabolites that mediate cross-kingdom communication (14, 15). Recent advances in metabolomics have discovered non-invasive biomarkers, therapeutic targets of fecal metabolites, and mechanistic insights into host-microbe interactions (16). NSP-rich diets induce gut dysbiosis and alter host metabolism. For instance, feeding a high-NSP diet (30% rice bran) significantly changes the composition of the cecal microbiome and reduces short-chain fatty acid production (17). Another study demonstrated that an NSP-rich challenge diet triggers inflammatory pathways in the jejunum and modulates the gut microbiota and metabolome of broilers (18). Pomegranate peel is rich in polyphenols known to modulate intestinal inflammation, but its anti-inflammatory mechanism in NSP-induced enteritis via the gut microbiota-metabolite axis remains largely unexplored. We therefore hypothesized that PPP supplementation would restore the NSP-induced imbalance of gut microbiota and key metabolites, thereby reducing intestinal inflammation and improving growth performance. Here, we induced intestinal inflammation in broilers using a 30% rice bran diet-a validated model of NSP-induced enteritis, and investigated the anti-inflammatory effects of pomegranate peel through microbial (16S rRNA sequencing), and fecal metabolites (untargeted metabolomics) analyses. This study reveals how pomegranate peel modulates the gut microbiota-metabolite axis to alleviate NSP-induced intestinal inflammation supporting its use as a natural feed additive for sustainable poultry production.

## 2 Materials and methods

### 2.1 Diets, animals, and feeding management

The research was conducted in accordance with guidelines established by China’s Ministry of Technology on Ethical Treatment for Laboratory Animals (398, 2006). The animal experiments were approved by the Institutional Animal Care and Use Committee (IACUC) of Heze University under protocol number 2025-001. A total of 288 one-day-old 817 crossbred broilers were obtained from Yunchen Jihe Poultry Breeding Co., Ltd. (Heze, China). The broilers were raised under carefully controlled environmental conditions, maintaining stable temperature, humidity, and proper airflow. For the first 3 days, the lighting system consisted of uninterrupted lighting, followed by a day cycle of 23 h of daylight and 1 h of darkness. During the first week, the ambient temperature was maintained at 33 ± 1 °C and was gradually reduced by 2–3 °C each subsequent week until reaching a stable temperature of 25 °C, which was then maintained until the conclusion of the experiment. Feed and water were provided *ad libitum*. Pomegranate peels were purchased from Hubei Huada Real Technology Co., Ltd. (Jingzhou, China). The PPP was prepared as described by Xu et al. (11). Dried pomegranate peels were ground, passed through a 60-mesh sieve, and kept in a ventilated container. The PPP contained approximately 15.61% tannic acid and 14.67% moisture. A total of 288 broilers were randomly assigned to six treatment groups, each with four replicates of 12 broilers. These groups were as follows: (1) Control group (CON), a basic diet, (2) NSP, fed with the basic diet of 30% of the rice bran, (3) 3PPP_NSP, fed with the basic diet of 30% of the rice bran, adding 3 g/kg of PPP, (4) 6PPP_NSP, fed with the basic diet of 30% of rice bran and 6 g/kg of PPP, (5) 12PPP_NSP, adding 30% of the rice bran to the basic diet plus 12 g/kg of PPP, and (6) 24PPP_NSP, adding 30% of the rice bran to the basic diet plus 24 g/kg of PPP.

### 2.2 Sample collection

Samples were obtained on days 7, 14, 21, and 28 of age, with six broilers randomly selected at each time point for sample collection. After blood sampling, the broilers were euthanized by cervical dislocation immediately. Subsequently, samples were collected from the duodenum, jejunum, and ileum (the duodenum, jejunum and ileum samples were obtained from the middle section of the intestinal). A portion of duodenum, jejunum and ileum tissue was fixed with 4% paraformaldehyde for histological evaluation, while the remaining tissue, along with serum and fecal materials, was stored at -80 °C for further analysis. Metabolomic profiling and 16S rDNA sequencing were conducted by Metware Biotechnology Inc (Wuhan, China).

### 2.3 Growth performance parameters

At ages of 7, 14, 21, and 28 days, following a 12 h feed withdrawal, the broilers were weighed and the food intake was recorded for each cage. For 1–7 days, 8–14 days, 15–21 days and 22–28 days, the average daily food intake (ADG), average daily feed intake (ADFI) and feeding-to-gain ratio (F/G) were computed.

### 2.4 Intestinal inflammation-mRNA expression levels in broilers

Total RNA was extracted from the jejunum samples (n=6) of each group at each time point in accordance with the manufacturer’s protocol using TRIzol reagent (Takara Biomedical Technology (Beijing) Co., Ltd). cDNA synthesis was performed using the Reverse Transcription Kit (Takara Biomedical Technology (Beijing) Co., Ltd). Primers specific for interleukin-1β (IL-1β), interleukin-6 (IL-6), tumor necrosis factor-alpha (TNF-α), and the reference gene β-actin were designed based on sequences from previous studies, as shown in **Supplementary Table 1** (19).

### 2.5 Histological observation

The collected tissues (n=6) of each group at each time point were fixed in 4% formaldehyde solution for 24 h at 4°C, then rinsed thoroughly with distilled water overnight. The samples underwent dehydration through a series of graded ethanol solutions, followed by clearing with dimethylbenzene, and were subsequently embedded in paraffin. A 4-μm thickness slice of jejunum was made by means of a rotating microscope, and then stained with hematoxylin and eosin. Measurements of villus height (VH), crypt depth (CD) were carried out at 4 x magnification (Leica, Germany). The tissue sections were mounted with neutral balsam (Tianjin Yongda, China) and observed under a microscopy imaging system (Nikon E100, Tokyo, Japan).

### 2.6 Microbial diversity and composition

Genomic DNA was taken from samples (n=6) in CON, NSP and 6PPP_NSP groups on days 14 using cetyltrimethylammonium bromide (CTAB) extraction, and their purity and concentration were assessed on a 1% gel by agarose gel electrophoresis. The DNA sample was diluted with sterile water to 1 ng/μL. Primers 515F (5 ‘-GTGCCAGCMGCCGCGGTAA-3’) and 806R (5 ‘-GGACTACHVGGGTWTCTAAT-3’) were used to amplify the V4 hypervariable region of 16S rRNA gene. Sequencing libraries were produced on an Illumina Nova Seq 6000 platform using Tru Seq DNA PCR-Free Sample Preparation Kit (Illumina, USA) to produce 250 base-pair paired-end reads. The raw data were processed through a pipeline of bio-informative methods: initial demultiplexing and quality filtering with FASTP (https://github.com/OpenGene/fastp), assembling an overlapping FLASH (http://ccb.jhu.edu/software/FLASH/), and removing the chimeric sequences by UCHIME against SILVA Reference Database (https://www.arb-silva.de/) Operative Taxonomic Units (OTUs) were grouped at 97% similarity using UPARSE (http://drive5.com/uparse/), and classification was done by using Mothur pipe according to SILVA database notes. The diversity analysis included the calculation of the alpha diversity indicators (Shannon, Simpson) and the beta diversity measure (the weighted UniFrac distances) via QIIME.

### 2.7 Untargeted metabolomics analysis of feces

400 μL (methanol: water = 7: 3, V/V) containing an internal standard was added to each sample (20 mg) from CON, NSP and 6PPP_NSP groups (n=6 per group) on days 14, and stirred for 3 min. The mixture is then subjected to ultrasonic treatment for 10 min in an ice bath, restirred for 1 min and then cooled for 30 min at 20 °C. The specimen was then centrifuged at a rate of 12,000 rpm for 10 min at 4 °C. The resultant sediment is removed and the supernatant is subjected to a second centrifugation at 12,000 rpm for 3 min at 4 °C, followed by an aliquot of 200 μL of the supernatants for LC-MS analysis. Using Waters ACQUITY Premier HSS T3 Tower (1.8 μm, 2.1 mm x 100mm), one aliquot was tested in positive ion mode, with 0.1% formic acid as solvent A and 0.1% formic acid in acetonitrile as solvent B in a gradient of between 5 and 20% of Solvent B for 2 min, rising to 60% in 3 min, then at 99% in one min, lasting for 90 s, and then back to 5% in 0.1 min, and sustained for 2.4 min. Mass spectrometric analysis was carried out with both positive and negative modes, scanning from m/z 75 to 1000 at a resolution of 35,000. Other MS parameters included 3.5 kV (positive) or 3.2 kV (negative), sheath gas 30 Arb, auxiliary gas 5, ion transfer tube temperature 320 °C, vaporizer temperature 300 °C, 30, 40 and 50 V collision energy, 1.00e-06 cps, top N set to 10 or top speed, and an exclusion duration 3 s. Raw data were converted to mzXML using ProteoWizard MSConvert (v3.0.8789) and processed with R XCMS (v3.12.0) for feature detection, alignment, and retention time correction (ppm=15, peakwidth=c (5, 30), mzdiff=0.01, method=centWave) (20, 21). Batch effects were corrected using QC samples, and metabolites with RSD >30% in QCs were removed.

The difference in the metabolism of the two groups was identified by differential variables (VIP) score (VIP > 1) and the *P* (<0.05, Student T Test). VIP scores were derived from the Orthogonal Partial Least Squares Discriminant Analysis (OPLS-DA), which also offered a score graph and a substitution test for overfitting evaluation. Prior to OPLS-DA, the log_2_ transform and mean alignment were used. To prevent overfitting, permutation testing with all permutations was performed. The identified metabolites have been annotated with the KEGG Complex Database (http://www.kegg.jp/kegg/compound/) and have been applied to map the metabolites to the KEGG pathway (http://www.kegg.jp/kegg/pathway.html).

### 2.8 Statistical analysis

Statistical evaluations were carried out with SPSS 23.0 software, incorporating one-way ANOVA followed by Tukey’s *post-hoc* multiple comparison procedures. Results are presented as means ± standard error (X ± SEM), with significance set at *P* > 0.05 for non-significant differences and *P* < 0.05 for significant differences. Graphical representations were created using Origin 9.0 (OriginLab, Northampton, MA).

## 3 Results

### 3.1 Effect of PPP on growth performance of NSP-challenged broilers

**Table 1** summarizes the impact of PPP on broiler growth performance in the presence of NSP challenge. The NSP challenge resulted in a significant reduction in body weight on days 7, 14, 21, and 28 as compared with CON (*P* < 0.05). There was no significant change in body mass between CON and PPP during the duration of the study (*P* > 0.05). A linear trend of increased body weight was observed with higher levels of PPP inclusion under NSP challenge conditions, although a reduction was noted at the 24PPP_NSP level, we speculated that high levels of PPP may synergize with high NSP to reduce growth performance. The NSP challenge significantly decreased the average daily gain from days 1–28 compared to the CON group (*P* < 0.05), although no significant differences in average daily gain were observed between the NSP and PPP _NSP groups (*P* > 0.05). Additionally, NSP challenge led to a reduction in average daily feed intake during days 8-14, 21-28, and from days 1–28 when compared to the CON group (*P* < 0.05). The 6PPP_NSP group exhibited an increase in average daily feed intake during days 1–7 and 15–21 relative to both the CON and NSP groups (*P* < 0.05). Moreover, supplementation with PPP at levels of 3, 6, 12, and 24 g/kg resulted in higher feed conversion ratios across all time periods compared to the CON group (*P*>0.05), with the 6PPP_NSP group showing results most similar to the CON group. Detailed VH and CD data was provided in **Supplementary Table 2**.

**Table 1:** Effect of dietary PPP supplementation on growth performance in broiler chickens challenged with NSP.

Items	Group						SEM	P-value
	CON	NSP	3PPP	6PPP	12PPP	24PPP		
Body weight (BW), g								
1d	47.29	51.66	47.67	50.08	46.42	44.87	1.65	0.083
7d	164.78	154.6	149.28	158.41	151.83	145.15	4.85	0.183
14d	368.53	324.58	337.03	362.56	351.45	323.39	11.56	0.068
21d	648.18^a^	538.19^c^	591.76^abc^	632.76^ab^	574.76^abc^	549.23^bc^	19.47	0.004
28d	1055.29^a^	841.08^b^	915.51^ab^	948.09^ab^	929.88^ab^	795.66^b^	31.43	<.001
Average daily gain (ADG), g								
1-7	19.58	17.16	16.93	18.05	17.57	16.71	0.85	0.309
8-14	33.96	28.33	31.29	34.03	33.27	29.71	2.19	0.475
15-21	46.61	35.60	42.46	45.03	37.22	37.64	3.39	0.146
21-28	67.85	50.48	53.96	52.55	59.19	45.24	5.39	0.091
1-28	36.00^a^	28.19^b^	30.99^ab^	32.07^ab^	31.55^ab^	26.81^b^	1.24	<.001
Average daily feed intake (ADFI), g								
1-7	20.87^b^	20.76^b^	21.70^a^	21.92^a^	20.29^b^	22.09^a^	0.17	<.001
8-14	36.00^b^	33.36^c^	36.00^b^	33.83^c^	35.95^b^	36.65^a^	0.14	<.001
15-21	53.50^bc^	52.60^c^	55.51^a^	55.86^a^	53.63^b^	53.15^bc^	0.22	<.001
21-28	90.87^a^	79.05^c^	74.74^d^	75.17^d^	82.22^b^	77.34^c^	0.48	<.001
1-28	50.29^a^	46.42^d^	47.00^cd^	46.69^cd^	48.07^b^	47.34^c^	0.16	<.001
Feed to gain ratio (F: G), g/g								
1-7	1.08	1.37	1.30	1.23	1.16	1.33	0.08	0.435
8-14	1.10	1.54	1.16	1.00	1.09	1.28	0.11	0.218
15-21	1.20	1.73	1.41	1.27	1.48	1.48	0.15	0.364
21-28	1.42	1.88	1.46	1.56	1.46	1.85	0.19	0.451
1-28	1.41^b^	1.72^ab^	1.52^ab^	1.47^ab^	1.54^ab^	1.84^a^	0.08	0.032

Body weight (BW), average daily gain (ADG), average daily feed intake (ADFI), and feed-to-gain ratio (F/G); Data are expressed as means ±pooled SEM (n =8). SEM- standard error of mean; a, b, c, d, means within a row with different superscripts indicate a significant difference (*P* <0.05).

### 3.2 Effect of PPP on inflammatory gene expression in the broiler jejunum

**Figure 1:**
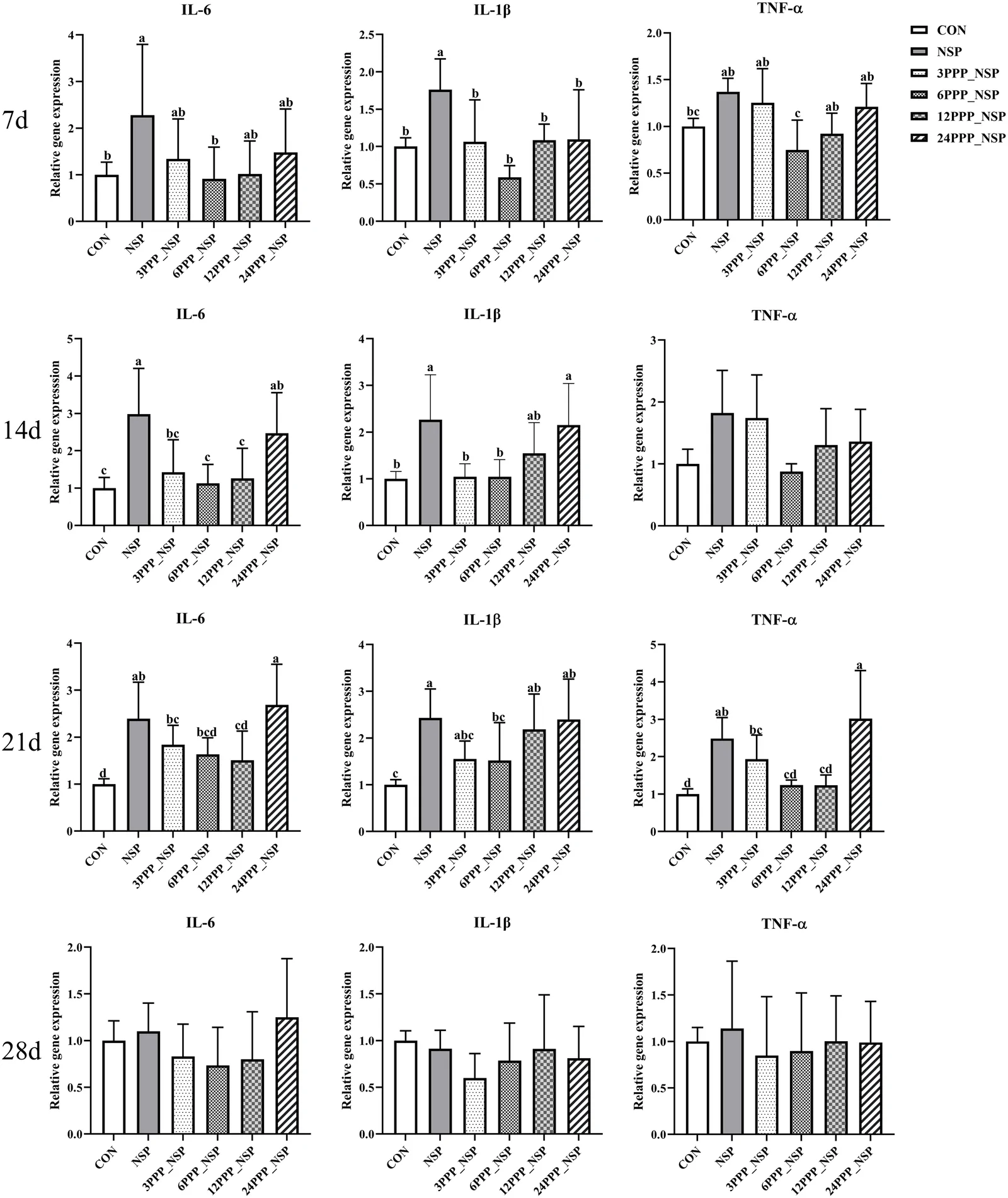
Effect of dietary PPP supplementation on inflammatory gene expression of jejunum in broiler chickens challenged with NSPs. The levels of inflammatory factors IL-6 (interleukin-6), IL-1β (interleukin-β), and TNF-α (tumor necrosis factor-α) at 7 days, 14 days, 21 days and 28 days of age; a, b, c, d, means within a row with different superscripts indicate a significant difference (*P* < 0.05).

The mRNA expression levels of inflammatory cytokines in the jejunum across all treatment groups are presented in **Figure 1**. On days 7, 14, and 21, IL-6 mRNA expression in the NSP group was significantly elevated (*P* < 0.05) compared with the CON group. Notably, IL-6 mRNA expression in the 6PPP**_**NSP group was significantly reduced (*P* < 0.05) on days 14 relative to the NSP group. Similarly, IL-1β mRNA expression was significantly increased (*P* < 0.05) in the NSP group on days 7, 14, and 21 compared to the CON group, whereas supplementation with PPP significantly downregulated IL-1β mRNA expression (*P* < 0.05) at the same time points. TNF-α mRNA expression was elevated in the NSP group on days 21 compared to the CON group (*P* < 0.05), dietary PPP at 6 g/kg and 12 g/kg effectively reversed the NSP-induced up-regulation of TNF-α expression (*P* < 0.05).

### 3.3 PPP alleviated NSP diet-induced intestinal damage in broilers

**Figure 2:**
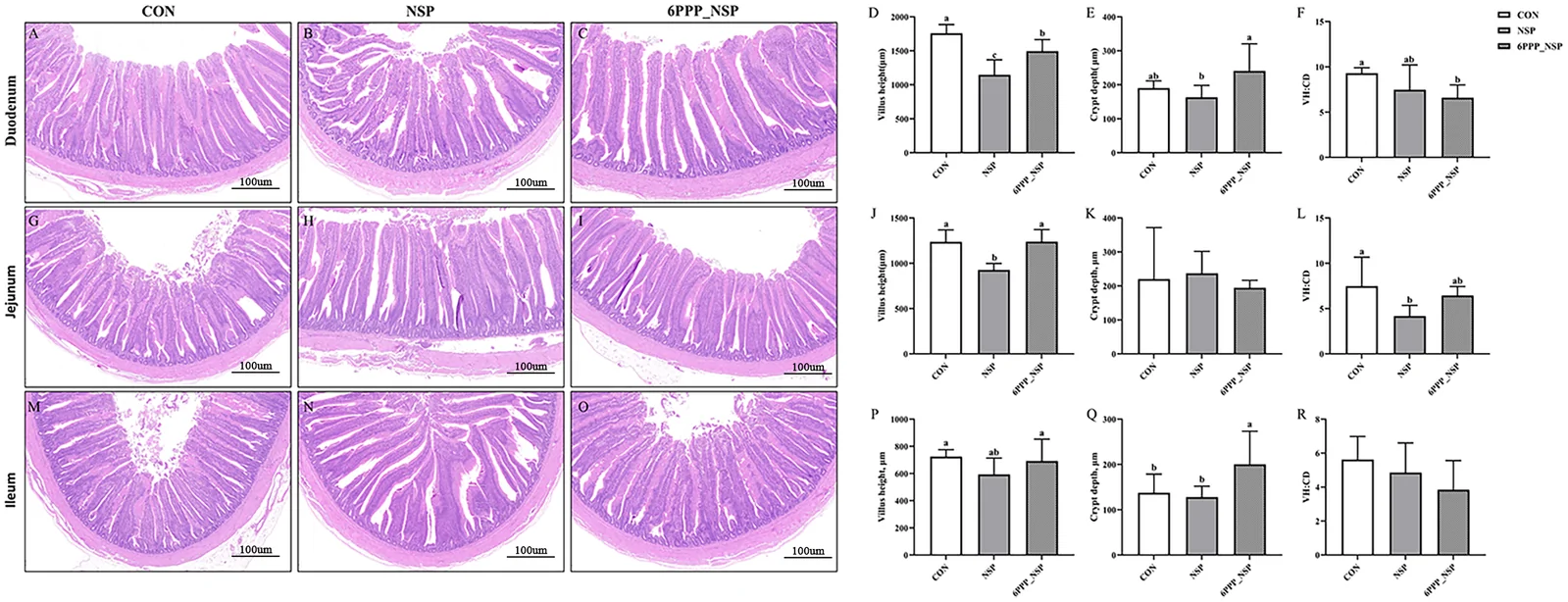
Effect of dietary 6PPP_NSP supplementation on intestinal morphology in broiler chickens challenged with NSPs. **(A–F)** duodenum; **(G–L)** jejunum; **(M–R)** ileum; a, b, c, d, means within a row with different superscripts indicate a significant difference (*P* < 0.05).

Based on the above findings, 6PPP_NSP group was selected for subsequent research (**Figures 2A–R**). Severe damage to the duodenal villi structure was observed at 14 days after NSP feeding, characterized by both villus height (VH) shortening and morphological alterations (**Figures 2A, B, D**). Consequently, the duodenal and jejunal VH, and the height of the villi compared to the crypt depth (VH:CD) were significantly decreased (*P*<0.05) in the NSP group compared to the CON group at 14 days post-NSP feeding **Figures 2G, H, J, L**). No significant changes in duodenal, jejunal and ileum crypt depth (CD) were observed up to 14 days after NSP administration (*P*>0.05) (**Figures 2E, K, Q**). Compared with the NSP group, the VH in the duodenum and jejunum of the 6PPP_NSP group was significantly elevated (*P*<0.05) (**Figures 2C, I, D, J**), while the VH in the ileum returned to normal levels (*P*>0.05) (**Figures 2M–P**). Simultaneously, compared with NSP, the VH:CD ratio in duodenum of the 6PPP_NSP group decreased significantly (*P*<0.05) (**Figures 2C, F**).

### 3.4 PPP attenuated gut dysbiosis in NSP-challenged broilers

**Figure 3:**
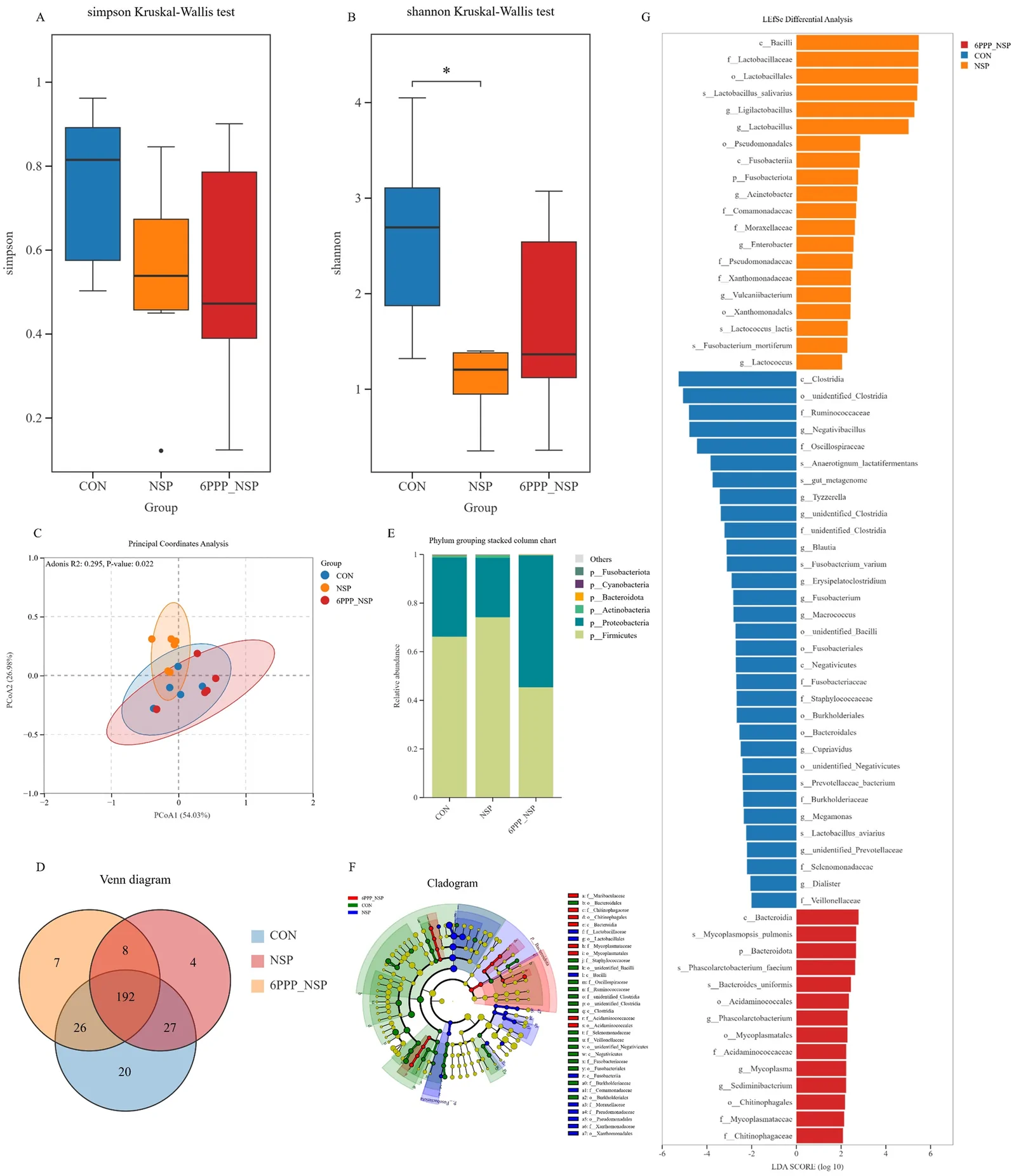
Effect of dietary 6PPP_NSP supplementation on feces microbiota diversity in broiler chickens challenged with NSPs. **(A)** Simpson index; **(B)** Shannon index; **(C)** Principal coordinates analysis plot; **(D)** Venn diagram of ASV/OTU across groups, the numbers in each section indicate the number of ASVs/OTUs contained in that section; **(E)** Microbial composition at the phylum level, the horizontal axis represents the grouping; the vertical axis indicates relative abundance; **(F)** Phylogenetic profile of specific bacterial taxa and predominant bacteria between the three groups. **(G)** histogram of Linear discriminant analysis (LDA), Substantially high bacterial taxa at different taxonomic levels (p = phylum, c = class, o = order, f = family, g = genus, and s = species) represented in CON with (blue), NSP (orange) and 6PPP_NSP **(red)** as indicated by LDA scores based on LEfSe. LDA score of 2.0 was used as a threshold for significance in LEfSe analyses; ^*^P < 0.05.

High-throughput 16S rDNA pyrosequencing was performed on the fecal samples to investigate the impact of PPP on the composition of intestinal flora. Alpha diversity metrics, such as the Shannon and Simpson indices, indicated a reduction in diversity following NSP treatment compared to the CON group (*P* < 0.05) (**Figures 3A, B**). After administering PPP, the microbial community’s diversity and evenness showed a slight recovery relative to the NSP group (*P*>0.05) (**Figures 3A, B**). Using weighted UniFrac principal coordinate analysis (PCoA), distinctions in species composition were observed between the NSP group and both the 6PPP_NSP and CON groups (**Figure 3C**), but not significant (*P*>0.05). The Venn diagram (**Figure 3D**) revealed 192 operational taxonomic units (OTUs) shared across all groups, while the CON, 6PPP_NSP, and NSP groups contained 20, 7, and 4 unique OTUs, respectively.

Analysis of microbiota composition across different taxonomic levels revealed that the predominant phyla in all three groups were *Firmicutes*, *Actinobacteria*, and *Proteobacteria*. At the phylum level, compared to the CON group, NSP treatment led to an increase in the relative abundances of *Firmicutes* and *Actinobacteria*, while the proportion of *Proteobacteria* decreased. These shifts were counteracted by PPP treatment (**Figure 3E**). To further identify taxa with significant differences among groups, linear discriminant analysis effect size (LEfSe) analysis was performed, with a threshold set at linear discriminant analysis (LDA) score greater than 2.0 and *P* < 0.05. The results showed that the *Phascolarctobacterium faecium*, *Bacteroides uniformis*, and members of the *Muribaculaceae* had higher LDA scores in the 6PPP_NSP group, indicating their increased abundance. Conversely, taxa including *Fusobacteriota* and *Fusobacterium mortiferum* exhibited higher LDA scores in the NSP group (**Figures 3F, G**).

### 3.5 PPP alters fecal metabolites in NSP-challenged broilers

**Figure 4:**
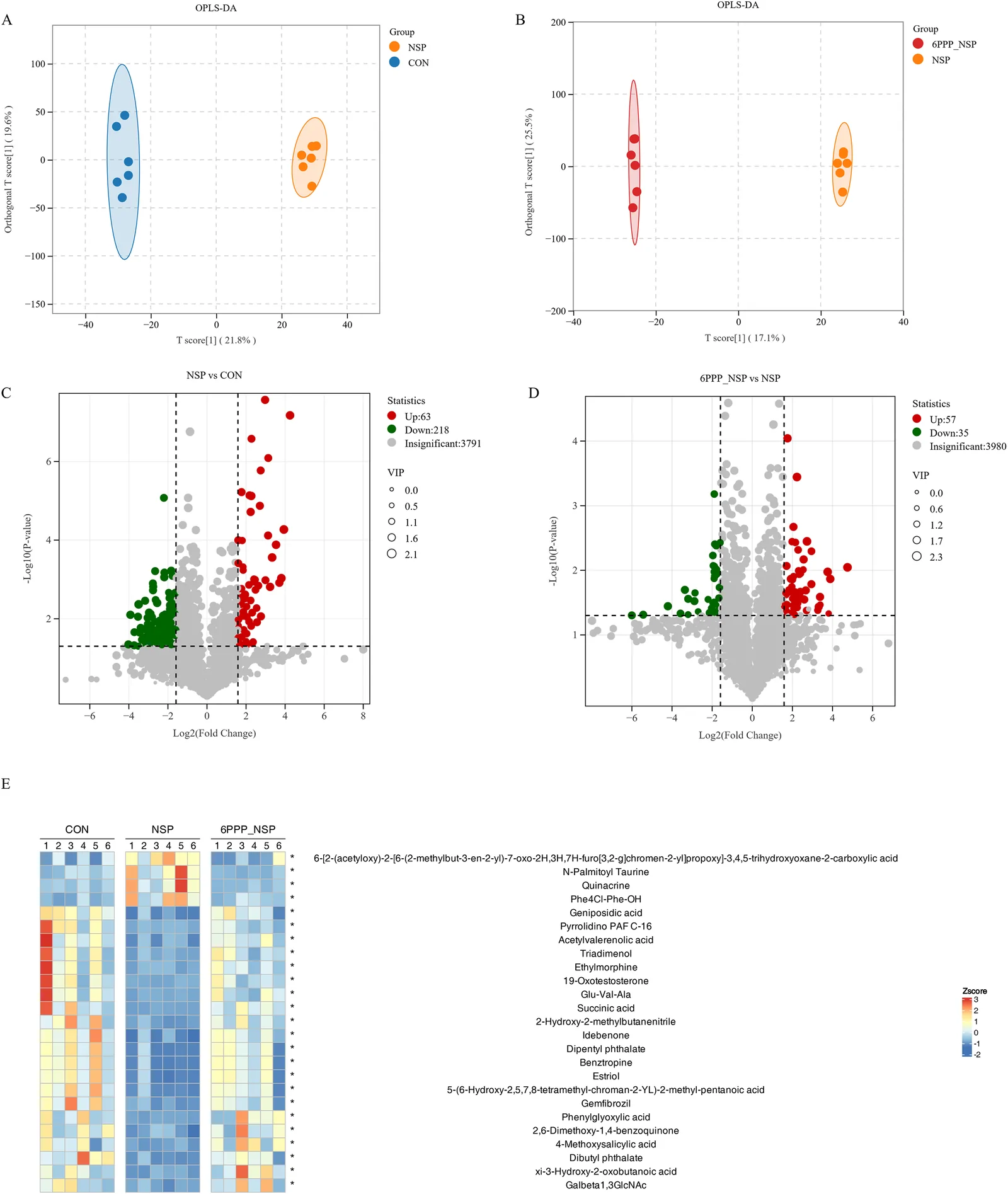
Fecal metabolomics analysis of the CON group, NSP group and 6PPP_NSP group. **(A, B)** respectively presents the OPLS-DA score situations between the NSP group and the CON group, as well as between the NSP group and the 6PPP_NSP group; **(C, D)** Volcanic plots situations between the NSP group and the CON group, as well as between the NSP group and the 6PPP_NSP group. **(E)** Heat map of metabolites whose abundance were altered by NSP compared with CON and then restored by PPP. ^*^P < 0.05.

Under both negative and positive ionization modes, a total of 2,023 and 2,722 metabolites were identified in the fecal samples. OPLS-DA was employed to differentiate the NSP vs CON group (R^2^Y of 0.997, Q^2^ of 0.818) and the 6PPP**_**NSP vs NSP (R^2^Y of 0.999, Q^2^ of 0.742) (**Figures 4A, B**). A good separation was observed between the groups, respectively. Metabolites with fold change > 3 or <1/3, *P*-value <0.05 and importance in projection (VIP) >1 were considered differential. Volcano plots were generated to visualize the metabolite differences between groups (**Figures 4C, D**). Compared with the CON group, 63 metabolites were significantly up-regulated and 218 metabolites were down-regulated in NSP group (*P* < 0.05). Compared with the NSP group, 57 metabolites were significantly up-regulated and 35 metabolites were down-regulated in 6PPP_NSP group (*P* < 0.05). Detailed information of the different metabolites is listed in **Supplementary Table 3**. In addition, PPP supplementation restored 25 metabolites change induced by NSPs (*P* < 0.05) (**Figure 4E**). For example, NSPs down-regulated the level of geniposidic acid by 0.22-fold compared the CON, while PPP restored it by 3.19-fold compared with the NSP group. Similarly, these metabolites also include succinic acid, 2,6-Dimethoxy-1,4-benzoquinone, and so on.

**Figure 5:**
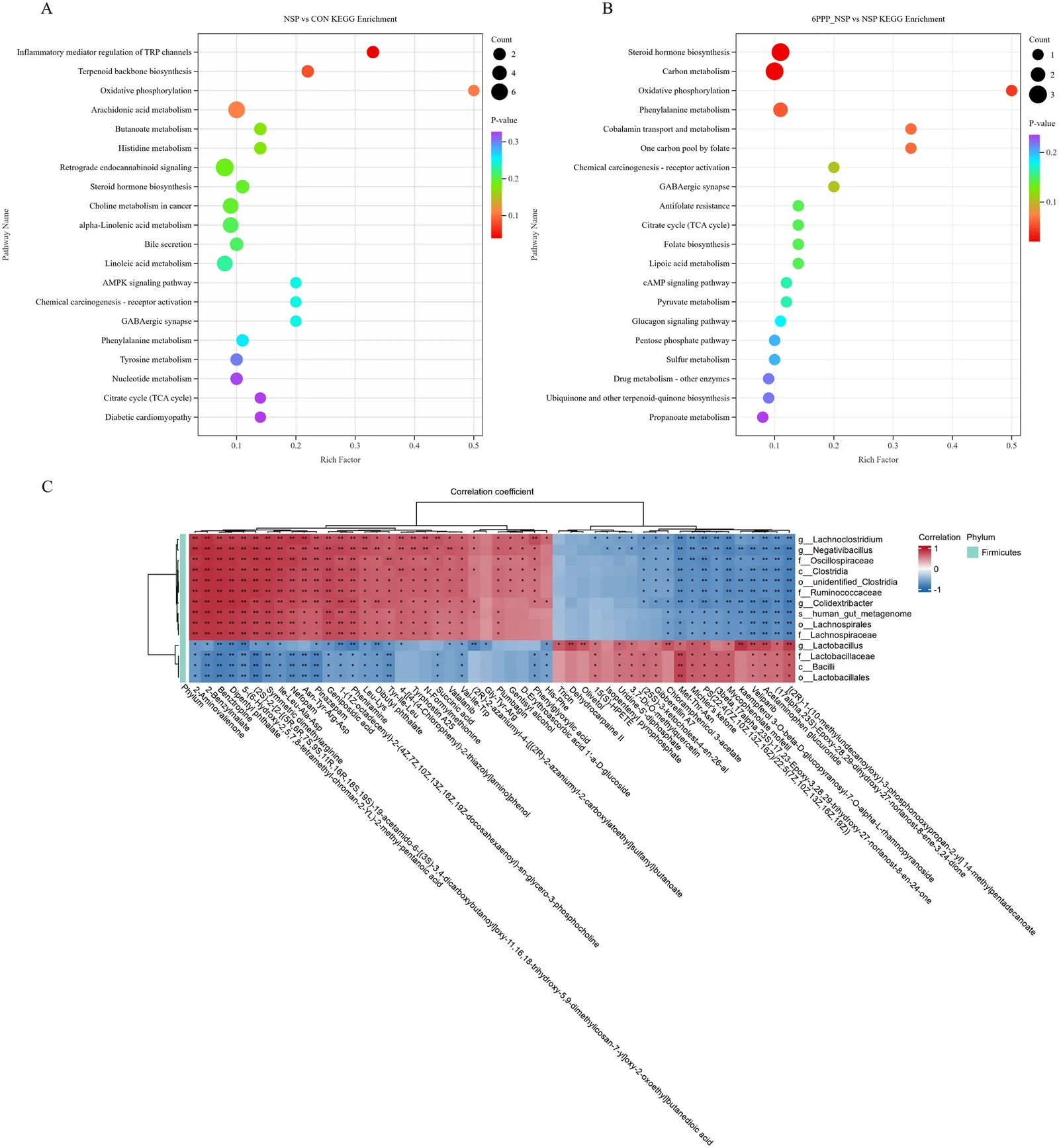
KEGG pathway enrichment analysis of differential metabolites, and correlation analysis of the gut microbiome and fecal metabolites between NSP group and the 6PPP_NSP group. **(A, B)** The KEGG enrichment analysis between the NSP group and the CON group, as well as between the NSP group and the 6PPP_NSP group. The horizontal axis represents the Rich Factor corresponding to each pathway, the vertical axis represents the pathway name, and the color of the dots reflects the *P*-value, with redder dots indicating more significant enrichment. The size of the dots represents the number of differentially enriched metabolites. **(C)** Correlation analysis. The horizontal axis represents metabolites, while the vertical axis represents the intestinal microbiota. Red color indicates a positive correlation, and blue color indicates a negative correlation. *P < 0.05, **P < 0.01 denoted statistical significance between bacterial taxa and metabolites.

These significantly different metabolites were subsequently mapped to KEGG metabolic pathways (top 20) for comparisons between NSP and CON (**Figure 5A**), 6PPP_NSP and NSP (**Figure 5B**). A comparison between the NSP and CON group revealed significant enrichment in pathways such as oxidative phosphorylation, inflammatory mediator regulation of TRP channels and terpenoid backbone biosynthesis. The comparison between 6PPP_NSP and NSP group showed marked enrichment in pathways including oxidative phosphorylation, phenylalanine metabolism, cobalamin transport and metabolism, one-carbon pool by folate, and carbon metabolism. The correlation between the differential fecal microbiota and metabolites in PPP treated broilers fed with NSPs were analyzed using Spearman’s rank correlation (**Figure 5C**). The study found that oxidative phosphorylation pathway related metabolite, succinic acid, was negatively correlated with *g_Lactobacillus* and positively correlated with *g_Lachnoclostridium*, *g_Negativibacillus*, *g_Colidextribacter*. The correlation between inflammatory factors (IL-6, IL-1β, TNF-α) and gut microbiota/metabolites were further calculated using Spearman’s rank correlation (**Supplementary Table 4**). Notably, tricin positively correlated with IL-6 and IL-1β. Indoleacetic acid was negatively correlated with all three cytokines. Additionally, *g_Megamonas* showed negative correlations with IL-6 and IL-1β.

## 4 Discussion

Although incorporating elevated amounts of NSPs in poultry diets can provide economic advantages by lowering costs and improving resource efficiency, its negative impacts on broiler performance and gastrointestinal health are extensively documented (3). Such as insoluble NSPs may lead to mechanically damage the intestinal mucosa, while soluble NSPs elevate digesta viscosity, creating a microenvironment conducive to the release of inflammatory mediators. Studies have shown that high NSP levels could increase digesta viscosity in the gastrointestinal tract, impair peristaltic efficiency, increase crypt depth, and induce villus atrophy, collectively compromising the intestinal barrier (5, 22). It has been confirmed that alternative feed components rich in NSPs, such as wheat, barley and rice bran, cause a decline in growth performance parameters (23, 24). Moreover, elevated NSPs intake up-regulates pro-inflammatory cytokines such as TNF-α, IL-1β, and IL-6 in broilers of jejunum, further exacerbating intestinal inflammation and negatively impacting nutrient absorption and growth performance (25). In this study, NSP group (high-NSP diet) exhibited marked intestinal damage, including villus atrophy, reduced VH, and VH/CD ratio. These morphological alterations were accompanied by a significant up-regulation of pro-inflammatory cytokines such as IL-6 and IL-1β. Collectively, these changes contributed to a decline in growth performance, as evidenced by reduced BW and ADFI, along with an increased F/G. The main components of herbal PPP (punicalagin, gallic acid, quercetin, and anthocyanins) have been shown to significantly suppress inflammatory responses by inhibiting inflammatory signaling pathways (26), enhancing antioxidant enzyme activity (27), and inhibiting the apoptosis of intestinal epithelial cells and maintain the integrity of villi structure (28). In this study, addition of PPP significantly alleviated the NSP-induced decline in growth performance and expression of intestinal inflammatory factors of broilers. We hypothesized that this effect may result from the synergistic action of bioactive compounds in PPP-such as ellagitannins (e.g., punicalagin), gallic acid, quercetin, and anthocyanins-through a multi-pathway mechanism involving inhibition of inflammatory signaling, suppression of pro-inflammatory cytokines (e.g., IL-6, TNF-α), and restoration of intestinal barrier integrity. Further research is needed to determine which specific component plays the main role.

In our study, we found that NSPs led to an increase in *Firmicutes* and *Actinobacteria*. *Firmicutes* mainly helped the host to assimilate energy from the diet (29). Studies have displayed that the relative abundance of the *Firmicutes* increases with age, and it is positively correlated with pro-inflammatory factors. Meanwhile, an increase in the *Firmicutes* can stimulate the expression of pro-inflammatory cytokines TNF-α, IL-1β, and IL-6 in colonic macrophages (30). *Actinobacteria* is a type of Gram-positive bacteria with high G+C content, widely distributed in soil, water bodies, and symbiotic systems of animals and plants (31). Although most *Actinobacteria* are renowned for their ability to produce antibiotics and perform symbiotic functions, some pathogenic *Actinobacteria* can directly or indirectly promote inflammatory responses by releasing virulence factors, activating host immune pathways, or inducing chronic infections (32). In addition, PPP reversed the increase in *Firmicutes* and *Actinobacteria* induced by NSP. It restored the intestinal balance and enhanced the intestinal homeostasis. Meanwhile, comparing with NSP, we found that PPP increased short-chain fatty acids (SCFAs)-producing genera such as *Phascolarctobacterium*, *Bacteroides uniformis*, and *Muribaculum*. SCFAs are important sources of energy in gut epithelium (33), and have the ability to adjust the immunity and the barrier function of the intestine, and restrain the pathogenic bacteria from adhering to the gut wall, thus avoiding inflammation (34). Moreover, SCFAs are not directly present in the diet. Instead, they are synthesized by symbiotic bacteria using the carbohydrates in the diet, and they are crucial for intestinal health (35). Furthermore, PPP can also reduce the increase of *Fusobacteriota* and *Fusobacterium mortiferum* in NSP group. *Fusobacteriota* may activate NF-κB and AKT/MAPK pathways to induce inflammatory cytokines, contributing to inflammatory bowel disease (IBD) and colorectal cancer (36). *Fusobacterium mortiferum* has been isolated from a human patient suffering from multibacterial sepsis (37) as well as from a mixed anaerobic infection of the thyroid gland (38). Typically considered a commensal organism in immunocompromised individuals, *Fusobacterium mortiferum* has been observed to disseminate beyond the intestinal tract, potentially leading to sepsis and an inflammatory response in the host (39). It is worth noting that PPP increased the relative abundance of *Proteobacteria* at the phylum level in our study. Increased *Proteobacteria* abundance is commonly considered a marker of dysbiosis and inflammation in many studies (40). This phylum includes well-known pathogens (e.g. some strains of *Escherichia coli*, *Salmonella*, *Campylobacter*) as well as commensal and even probiotic members. For instance, *E. coli Nissle 1917* is a classic probiotic strain that improves intestinal barrier function, enhances immune responses and reduces pathogen colonization in poultry (41). In addition, consistent with our research, Khan et al. also showed that probiotic supplementation could increase phylum-level *Proteobacteria* and improve growth performance and egg quality (42). Thus, we hypothesize that the phylum-level increase in Proteobacteria following PPP supplementation might be reasonable, reflecting an expansion of beneficial commensals rather than pathobionts. Further studies are needed to identify which specific bacteria taxa within this phylum were altered.

Gut metabolites, produced jointly by the host and the gut microbiota, are critical for supporting host health (43). Based on the KEGG metabolic pathway analysis, we found that several pathways, such as oxidative phosphorylation, citrate cycle, were involved in both NSP vs CON and 6PPP_NSP vs NSP. The metabolite relate to these pathways is succinic acid. Oxidative phosphorylation is the primary metabolic pathway for energy production, playing a key role in animal growth and development (44). Succinic acid is situated at the core position of various metabolic pathways, such as TCA cycle and oxidative phosphorylation, and can be produced by glycolysis and fatty acid β-oxidation pathways, as well as by intestinal microbial fermentation of dietary fiber (45). Recently, numerous studies have found that succinic acid is related to growth performance. For instance, 0.4% succinic acid supplementation reduced average daily feed intake, blood glucose, and triglycerides in broilers, while increasing high-density lipoprotein cholesterol, enhancing propionic acid content in the caecum, and altering gut microbiota composition (46). The inclusion of 1% succinic acid in pig feed significantly increased average daily gain, improved feed-to-gain ratio, enhanced eye muscle area and fat content, while simultaneously improving meat quality (47). Succinic acid enhanced mitochondrial activity by upregulating the expression of TCA enzymes and oxidative phosphorylation pathway (48). Moreover, succinic acid, is a key microbial metabolite and metabolic intermediate, and has been shown to activate the SUCNR1 receptor on intestinal tuft cells, thereby triggering the IL-25/ILC2/IL-13 signaling cascade. This pathway enhances goblet cell secretion of RELMβ mucin, strengthens the mucosal barrier from a physical perspective, and induces macrophage polarization toward the anti-inflammatory M2 phenotype, collectively reducing pro-inflammatory cytokine release and ameliorating intestinal inflammatory conditions such as IBD (49). Consistent with the present study, in this study, NSPs reduced succinate content compared with the CON group (0.11-fold), whereas PPP treatment significantly increased it by 6.62-fold compared with the NSP group. We therefore hypothesized that PPP may enhance broiler growth performance under NSPs induction by upregulating succinate levels via the TCA cycle and oxidative phosphorylation pathways. In addition, PPP significantly increased levels of bioactive compounds capable of effectively suppressing inflammation, such as 2,6-dimethoxy-1,4-benzoquinone (2,6-DMBQ) and geniposidic acid (GPA). 2,6-DMBQ may exert its anti-inflammatory effects by inhibiting the activation of the key inflammasome, NLRP3 (50). Similarly, as a cycloartenol glycoside, GPA inhibits the activity of the IκB kinase (IKK) complex in a lipopolysaccharide-induced inflammatory model by blocking the signal transduction of TLR4 and its downstream adaptor molecule MyD88, thereby preventing the phosphorylation and degradation of IκBα (51–53). By maintaining the stability of IκBα, GPA significantly reducing the expression of pro-inflammatory factors such as TNF-α, IL-6 and IL-1β (54). This is similar to the mechanism by which punicalagin and ellagic acid found in pomegranate peel alleviate inflammation. For example, ellagic acid ameliorates lung injury and inflammation by blocking IκBα degradation and phosphorylation, thus inhibiting NF-κB activation (55). Zeng et al. also reported that punicalagin significantly reduced the expression of phosphorylated NF-κB p65, IκBα and p38 in lung tissue, as well as the pro-inflammatory cytokines TNF-α and IL-6 in lipopolysaccharide (LPS)-induced acute lung injury in mice (56). Furthermore, it is interesting to note that a dietary intake of 600 mg/kg GPA increased weight gain and feed conversion efficiency, whilst also improving meat quality (57). In this study, NSPs reduced the levels of 2,6-DMBQ and GPA compared with the CON group, whereas treatment with PPP significantly increased their levels; we therefore hypothesized that PPP may alleviate NSP-induced intestinal inflammation in broiler chickens by increasing the levels of 2,6-DMBQ and GPA, which in turn block IκBα degradation and phosphorylation, thereby inhibiting NF-κB activation.

## 5 Conclusions

PPP can alleviate the inflammatory response and intestinal damage induced by NSPs, and promote the growth of broilers by strengthening the intestinal barrier and reducing the levels of inflammatory factors. Moreover, PPP could restore the imbalance of intestinal flora and regulating the metabolism of SCFAs in intestinal inflammation in broilers caused by NSPs. These findings enhance the protective effect of PPP under NSPs challenge and provide new insights and potential regulatory strategies. Nevertheless, the precise effective dose of individual bioactive compound in PPP required to alleviate NSP-induced intestinal inflammation remains to be determined. In addition, further investigations are warranted to validate the proposed signaling cascades at the protein level, and evaluate the long-term safety, large-scale feasibility, and cost-effectiveness of PPP before its practical application in commercial poultry production.

## Data Availability

The datasets presented in this study can be found in online repositories. The names of the repository/repositories and accession number(s) can be found in the article/**Supplementary Material**.
