# Supplementary material for: Integrated gut microbiota and metabolomics analysis of the effect of pomegranate peel powder on intestinal inflammation in broilers induced by high non-starch polysaccharides
Source: Front Immunol. 2026 Aug 12;17:1857398. doi: 10.3389/fimmu.2026.1857398 (PMC13506736; doi:10.3389/fimmu.2026.1857398)
Supplement: Supplementary file 1 [file SupplementaryFile1.docx]

**Supplementary Table 1**. Sequences of primer pairs used for amplification of target and reference genes

| **Gene** |  | Primer (5′–3′) | Gene BankAccession Number |
| --- | --- | --- | --- |
| proinflammatory cytokines | IL-6[16] | CTGTTCGCCTTTCAGACCTACC GACCACTTCATCGGGATTTATCA | HM179640.1 |
|  | IL-1β[17] | GGTCAACATCGCCACCTACA CATACGAGATGGAAACCAGCAA | XM_015297469.3 |
|  | TNF-α[18] | TGTATGTGCAGCAACCCGTA CCACACGACAGCCAAGTCAA | NM_204267.2 |
| housekeeping gene | β-Actin[16] | CCTGGCACCTAGCACAATGAA GGTTTAGAAGCATTTGCGGTG | NM_205518.2 |
