# Supplementary material for: Integrated gut microbiota and metabolomics analysis of the effect of pomegranate peel powder on intestinal inflammation in broilers induced by high non-starch polysaccharides
Source: Front Immunol. 2026 Aug 12;17:1857398. doi: 10.3389/fimmu.2026.1857398 (PMC13506736; doi:10.3389/fimmu.2026.1857398)
Supplement: Supplementary file 2 [file SupplementaryFile4.docx]

**Supplementary Table S4** Spearman association analysis between inflammatory factors and gut microbiota, metabolites.

| Differential metabolites and bacterial genera | Inflammatory factors | Correlation | P-value |  |
| --- | --- | --- | --- | --- |
| *Sucrose* | IL-6 | 0.263 | 0.291 |  |
|  | IL-1β | 0.618 | 0.006 | **﹡﹡ |
|  | TNF-α | 0.179 | 0.478 |  |
| *7-Methoxycoumarin* | IL-6 | 0.329 | 0.182 |  |
|  | IL-1β | 0.595 | 0.009 | ** |
|  | TNF-α | 0.449 | 0.062 |  |
| *Tricin* | IL-6 | 0.674 | 0.002 | ** |
|  | IL-1β | 0.608 | 0.007 | ** |
|  | TNF-α | 0.437 | 0.070 | * |
| *Ozagrel* | IL-6 | -0.395 | 0.104 |  |
|  | IL-1β | -0.496 | 0.036 | * |
|  | TNF-α | -0.585 | 0.011 | * |
| *(R)-3-Amino-3-phenylpropanoic acid* | IL-6 | -0.587 | 0.010 | * |
|  | IL-1β | -0.618 | 0.006 | ** |
|  | TNF-α | -0.515 | 0.029 | * |
| *Corymboside* | IL-6 | 0.562 | 0.015 | * |
|  | IL-1β | 0.426 | 0.078 |  |
|  | TNF-α | 0.538 | 0.021 | * |
| *Aspartate semialdehyde* | IL-6 | -0.472 | 0.048 | * |
|  | IL-1β | -0.139 | 0.581 |  |
|  | TNF-α | -0.418 | 0.084 |  |
| *Spectinomycin* | IL-6 | -0.344 | 0.163 |  |
|  | IL-1β | -0.220 | 0.381 |  |
|  | TNF-α | -0.531 | 0.023 | * |
| *Lys-Glu-Gly* | IL-6 | -0.480 | 0.044 | * |
|  | IL-1β | -0.408 | 0.093 |  |
|  | TNF-α | -0.292 | 0.240 |  |
| *N-Phthalyl-L-tryptophan* | IL-6 | 0.267 | 0.284 |  |
|  | IL-1β | 0.678 | 0.002 | ** |
|  | TNF-α | 0.352 | 0.152 |  |
| *Gly-Phe-Asp* | IL-6 | -0.501 | 0.034 | ﹡ |
|  | IL-1β | -0.445 | 0.064 |  |
|  | TNF-α | -0.075 | 0.766 |  |
| *Phe-Gln-Leu* | IL-6 | -0.352 | 0.152 |  |
|  | IL-1β | -0.573 | 0.013 | * |
|  | TNF-α | -0.418 | 0.084 |  |
| *Ile-Leu-Lys* | IL-6 | -0.527 | 0.025 | * |
|  | IL-1β | -0.484 | 0.042 | * |
|  | TNF-α | -0.416 | 0.086 |  |
| *TyrMe-Lys-OH* | IL-6 | -0.511 | 0.030 | * |
|  | IL-1β | -0.418 | 0.084 |  |
|  | TNF-α | -0.261 | 0.295 |  |
| *Indoleacetic acid* | IL-6 | -0.631 | 0.005 | ** |
|  | IL-1β | -0.536 | 0.022 | * |
|  | TNF-α | -0.521 | 0.027 | * |
| *6-C-beta-glucopyranosyl-8-C-alpha-arabinopyranosylapigenin* | IL-6 | 0.364 | 0.137 |  |
|  | IL-1β | 0.228 | 0.363 |  |
|  | TNF-α | 0.472 | 0.048 | * |
| *3,7-Di-O-methylquercetin* | IL-6 | 0.511 | 0.030 | * |
|  | IL-1β | 0.488 | 0.040 | * |
|  | TNF-α | 0.406 | 0.095 |  |
| *Pinazepam* | IL-6 | -0.426 | 0.078 |  |
|  | IL-1β | -0.664 | 0.003 | ** |
|  | TNF-α | -0.575 | 0.013 | * |
| *Versicolorin B* | IL-6 | -0.366 | 0.135 |  |
|  | IL-1β | -0.666 | 0.003 | ** |
|  | TNF-α | -0.286 | 0.250 |  |
| *5-Oxo-4,5-dihydro-2-furylacetic acid* | IL-6 | 0.600 | 0.009 | ** |
|  | IL-1β | 0.581 | 0.011 | * |
|  | TNF-α | 0.381 | 0.119 |  |
| *2-Methoxycinnamic acid* | IL-6 | 0.296 | 0.233 |  |
|  | IL-1β | 0.618 | 0.006 | ** |
|  | TNF-α | 0.373 | 0.128 |  |
| *(r)-Methylmalonate semialdehyde* | IL-6 | -0.195 | 0.438 |  |
|  | IL-1β | -0.461 | 0.054 |  |
|  | TNF-α | -0.624 | 0.006 | ** |
| *3-Oxooctadecanoic acid* | IL-6 | 0.503 | 0.034 | * |
|  | IL-1β | 0.193 | 0.443 |  |
|  | TNF-α | 0.511 | 0.030 | * |
| *g__unidentified_Prevotellaceae* | IL-6 | -0.431 | 0.074 |  |
|  | IL-1β | -0.071 | 0.779 |  |
|  | TNF-α | -0.469 | 0.050 |  |
| *g__Sediminibacterium* | IL-6 | -0.286 | 0.250 |  |
|  | IL-1β | -0.151 | 0.550 |  |
|  | TNF-α | -0.475 | 0.046 | * |
| *g__Lactobacillus* | IL-6 | 0.329 | 0.182 |  |
|  | IL-1β | 0.086 | 0.735 |  |
|  | TNF-α | 0.527 | 0.025 | * |
| *g__Megamonas* | IL-6 | -0.474 | 0.047 | * |
|  | IL-1β | -0.552 | 0.018 | * |
|  | TNF-α | -0.434 | 0.072 |  |
| *g__Acinetobacter* | IL-6 | 0.476 | 0.046 | * |
|  | IL-1β | 0.331 | 0.179 |  |
|  | TNF-α | 0.432 | 0.073 |  |

*Correlation was significant at the 0.05 level, **Correlation was significant at the 0.01 level.
